# Estimation of Risk of Recurrence and Toxicity Among Oncologists and Patients With Resected Breast Cancer: A Quantitative Study

**DOI:** 10.3389/fpsyg.2020.540083

**Published:** 2020-10-27

**Authors:** Laura Ciria-Suarez, Paula Jimenez-Fonseca, Raquel Hernández, Jacobo Rogado, Caterina Calderon

**Affiliations:** ^1^Department of Clinical Psychology and Psychobiology, Faculty of Psychology, University of Barcelona, Barcelona, Spain; ^2^Department of Medical Oncology, Central University Hospital of Asturias, Oviedo, Spain; ^3^Department of Medical Oncology, Canary University Hospital, Santa Cruz de Tenerife, Spain; ^4^Department of Medical Oncology, Infanta Leonor University Hospital, Madrid, Spain

**Keywords:** breast cancer, chemotherapy, prognosis, shared decision making, toxicity

## Abstract

Shared decision-making regarding adjuvant systemic therapy in breast cancer is based on both properly conveying information about the prognosis of the disease and the benefits and risks of adjuvant treatment, as well as the patient’s ability to understand this information. This work proposed to analyze oncologists’ and patients’ perceptions of the risk of recurrence with and without chemotherapy and toxicity, and the factors influencing said impressions. This was a prospective, cross-sectional, multicenter study that involved 281 breast cancer patients and 23 oncologists. Prognosis (risk of recurrence with and without chemotherapy and risk of severe toxicity with chemotherapy) and shared decision making (SDM) questionnaires were completed by all participants; breast cancer patients also filled out the 18-item Brief Symptom Inventory (BSI-18). Oncologists’ prediction of risk of relapse without and with chemotherapy (30.4 and 13.3%) and risk of severe toxicity (9.8%) were more optimistic than those of breast cancer patients (78.6, 29.6, and 61%, respectively). The greater the severity, the higher the risk of relapse according to the oncologists (*p* = 0.001); not so for the patients. Older physicians and more experienced ones predicted lower risk of relapse with and without chemotherapy and less severe toxicity than younger doctors and those with less experience (*p* < 0.001). Oncologists’ SDM and their prediction of risk of relapsing with chemotherapy correlated negatively with patients’ SDM and their prediction of risk of severe toxicity (*p* < 0.01). There is a positive correlation between psychological distress (BSI-18) and prognosis of risk of recurrence with chemotherapy in breast cancer patients (*p* < 0.001). These results stress the importance of improving doctor–patient communication in SDM. In breast cancer patients undergoing treatment with curative intent, expectations of being cured would increase and treatment-related anxiety would decrease by enhancing doctor–patient communication to coincide more with respect to risk of relapse and toxicity, thereby enhancing patients’ quality of life.

## Introduction

Breast cancer is the second most common cancer overall and the leading cancer in women, with more than two million new cases diagnosed in 2018 (Fitzmaurice et al., 2018). Surgery is the treatment of choice for non-advanced breast cancer; chemotherapy, radiotherapy, and hormone therapy are adjuvants with consolidated benefit in diminishing the risk of relapse and improving long-term survival (Waks and Winer, 2019). These treatments provoke fear surrounding side effects such as alopecia, body changes, pain, fatigue… (Dooley et al., 2017) that add to their fear of recurrence (Miroševič et al., 2019). In recent years, other factors, such as tumor grade, estrogen receptor, and progesterone receptor status, HER2 overexpression, and, in some cases, genomic profiles, have been added to the prognostic value of TNM staging based on tumor size (T), number of regional lymph nodes affected (N), and the presence or absence of distant metastases (M), yielding more robust and precise prognostic information (Schönherr et al., 2012; Piñeros et al., 2019). Physicians estimate risk of adjuvant treatment-associated toxicity on the rates of side effects reported in clinical trials. Nevertheless, these trials are conducted in a highly selected population; thus, their data can scarcely be extrapolated to clinical practice where patients are more fragile and older and present more comorbidities, all of which can modify patients’ risk of toxicity (Schönherr et al., 2012; Piñeros et al., 2019). Knowing the prognosis of the disease and risk of adjuvant treatment-related toxicity is relevant to doctor–patient shared decision making (SDM) and tailoring treatment to risk, as well as helping the patient to cope better and lessening psychological distress (Lobb et al., 2001). Nevertheless, little is known about the effectiveness of communicating this kind of information in the oncologist’s office in a situation in which the effect of recent cancer surgery and emotional stress affect the ability to comprehend the language being used, thereby casting doubt on the validity of this SDM between patient and oncologist as to the advisability of receiving adjuvant treatment, its benefits, and risks. Furthermore, several factors influence how the patient participates in SDM (Street et al., 1995; Bakker et al., 2001; Janz et al., 2004; Maly et al., 2004; Ingersoll et al., 2019). Low-educated and older patients are often associated with a passive role (Street et al., 1995), with education being more consistent than age as a factor impinging on patient–physician communication (Janz et al., 2004). Additionally, doctors may provide less informative to individuals with lower levels of education (high school or less) and lower incomes (Bakker et al., 2001). As for race, fewer consultations for information concerning prognosis have been reported in the black and Latino populations, as well as unrealistic optimism regarding this prognosis (Ingersoll et al., 2019). In North American studies, ethnic minority women (i.e., African American and Latino breast cancer patients) were considerably less likely than white patients to perceive themselves as the chief treatment decision-makers, while at the same time, they were more likely to question their physician about their treatment, possibly due to mistrust of the healthcare system (Maly et al., 2004).

Most of the literature comparing doctors’ and patients’ expectations is founded on research in more advanced stages of the disease, concluding that patients tend to be more optimistic than doctors (Robinson et al., 2008; Gramling et al., 2016; Malhotra et al., 2019). Few publications address this issue in early-stage breast cancer (Siminoff et al., 1989; Ravdin et al., 1998). The few studies found reveal that agreement between patients and oncologists regarding the benefits and risks involved with adjuvancy is poor (Siminoff et al., 1989) and that patients are apt to overestimate the curative value of adjuvant therapy in 70–80% of the cases with respect to non-treatment (Ravdin et al., 1998). The optimistic confidence in treatment may reflect trouble in physician–patient communication (Mackillop et al., 1988), the influence of other sources of information (Adamson et al., 2019), or patients’ minimizing or denying that cancer can be life-threatening (Mackillop et al., 1988; Wakiuchi et al., 2019).

Coincidence between patients and oncologists in estimating risk of disease relapse and chemotherapy-associated toxicity is important to reduce confusion, adjust patients’ expectations, boost treatment compliance and involvement in SDM, and plan medical care and healthcare services that patients may need, thereby improving their quality of life (Lobb et al., 1999). So far as we know, in the setting of resected, non-metastatic breast cancer, there are no recent studies that examine doctor–patient coincidence with respect to the estimation of relapse with/without adjuvant chemotherapy and regarding the risk of treatment toxicity, which is the main objective of our study. Secondarily, we seek to explore the sociodemographic and psychological factors associated with estimating risk of recurrence and toxicity. We postulate that oncologists will be more realistic in their estimations of these two situations than patients and that the greater the patient-perceived risk of relapse and treatment-related toxicity, the less their satisfaction with SDM.

## Methods

### Study

This is a prospective, cross-sectional, multicenter study involving oncologists and non-metastatic breast cancer patients from all over Spain. Inclusion criteria were as follows: >18 years of age; histologically confirmed, non-advanced, resected solid tumor; eligible for adjuvant treatment. Exclusion criteria consisted of adjuvant treatment with hormone therapy alone and patients with dementia or any other serious mental illness that, in the investigator’s opinion, impeded survey comprehension or hampered the patient’s ability to participate in the study. Of the 310 breast cancer patients screened, 29 were ineligible (5 failed to meet inclusion criteria, 9 met an exclusion criterion, and 15 had incomplete data).

### Ethics Statements

The study was approved by the Research Ethics Committee of the Principality of Asturias (January 19, 2015) and by the Spanish Agency of Medicines and Medical Devices (AEMPS) (April 4, 2015). All participants signed an informed consent form prior to participation. Participation was voluntary and anonymous and would not affect patient care. Subjects completed questionnaires following their first appointment with the oncologist, approximately 1 month after surgery. The physician had informed them of the risk of relapse with and without treatment suited to their stage of cancer, risk factors, molecular subtype, and possible side effects of chemotherapy. Data were collected and updated by medical oncologist, specifically trained to meet the study requirements, through a web-based platform.^1^

### Data and Questionnaires

Sociodemographic and clinical data were collected during the first visit to the medical oncology department during which the advisability of receiving adjuvant treatment was discussed. Oncologists had to indicate their age and years of experience, and both clinicians and patients had to predict risk of relapse with and without chemotherapy and the risk of toxicity using a 0- to 100-point numerical rating scale. Participants completed questionnaires [nine-item Shared Decision Making Questionnaire (SDM-Q-9) and 18-item Brief Symptom Inventory (BSI-18)] at home between the first visit and the start of adjuvant chemotherapy and oncologists filled in the SDM-Q-Doc after the first consultation.

Doctors had to answer the SDM Questionnaire-Physician’s version (SDM-Q-Doc) (Scholl et al., 2012) to express their perspective on SDM and how well they followed it with their patients. The questionnaire consists of nine items, each of which describes one step of the SDM process; it was adapted and subsequently validated with good internal consistency in Spain (α = 0.90) (Calderon et al., 2017). Clinicians had to rate each item on a five-point Likert scale, yielding a summary score from 0 to 36; the higher the score, the greater the physician’s satisfaction with the information provided.

Participants completed the SDM Questionnaire–Patient’s Version (SDM-Q-9). This questionnaire was published by Kriston et al. (2010), adapted to Spanish, and has proven good internal consistency in Spain (α = 0.90) (Calderon et al., 2018). It consists of nine items, each portraying one step of the SDM process (Kriston et al., 2010), and is appraised on a five-point Likert scale with a summary score of 0–36; the higher the score, the greater the patient’s satisfaction with the information received.

Patients also answered the BSI-18 (Derogatis, 1993) that contains 18 items assessing somatization, depression, and anxiety. Respondents were asked to answer based on how they had felt over the previous 7 days; each item was rated on a five-point Likert scale. Participants whose *T*-score was ≥67, as per cutoff values recommended by Derogatis (1993), were deemed “probable anxiety or depression.” The higher the score, the greater the psychological distress in the previous month. α coefficients ranged between 0.75 and 0.88 in Spain sample (Calderon et al., 2020).

### Statistical Methods

We used descriptive statistics for breast cancer patients’ clinical data, as well as for participants’ and oncologists’ demographic information. *T*-test was used for quantitative variables and χ^2^ test for qualitative variables to appraise the degree of contrast between prognosis and toxicity estimations made by patients and oncologists. We used analyses of variance to assess variations in doctors’ and patients’ estimation by stage and age; Pearson correlation coefficients analyzed the relationships between estimations and demographic variables, SDM, and psychological distress. Given the large study sample size, the *F* ratio could incorrectly detect statistically significant differences. We therefore included the effect size (η^2^) using the classic formula proposed by Fisher (1928) to adjust effect estimate, avoiding bias sample. The interpretation of effect size is straightforward: values <0.20 indicate a very small or insignificant magnitude of effect; 0.20–0.49, a small effect size; 0.50–0.79, a moderate effect size; and values >0.80 indicate a large effect size. We set a statistical significance level of 0.05. Statistics were performed using the IBM SPSS software package for Windows, version 23.0 (SPSS, Inc., Chicago, IL).

## Results

### Participants’ Characteristics

The physicians’ sample comprised 23 medical oncologists (5 males and 13 females) employed at 11 Spanish hospitals. They have a mean age of 36.3 years (SD = 8.1, range = 28–59 years) and 12.9 years (SD = 9.8, range = 4–38 years) of experience, respectively. Sixty-five percent are super-specialists, and 56.5% work at a public university hospital.

The breast cancer patient sample consisted of 281 subjects. Cancers were stage IB (38.4%), II (52.3%), or III (9.3%). All subjects had undergone curative surgery in the previous month (53.5% partial resection vs. 46.5 complete resection) and were treated with adjuvant chemotherapy (64.2% conventional and 35.8% modified treatment), and 66.4% received sequential radiotherapy.

Mean age was 53.2 years (SD = 10.8, range = 26–77); 72% were married or partnered; 77.8% had primary or secondary education, and 44.4% were retired.

### Estimation of Risk of Recurrence and Toxicity

Oncologists’ prognosis of risk of breast cancer relapse without chemotherapy was 30.4% (45.3% deemed risk to be low), while the estimated risk of recurrence with chemotherapy-related chemotherapy was 13.3% (91.4% calculated that it would be very low). The risk of severe toxicity was estimated to be 9.8% (96.6% of the oncologists thought it was very low) (see Table 1).

**TABLE 1:** Prognostic predictions about risk of relapse and toxicity.

Variables	M (SD)	Very low	Low	High	Very high
Oncologists’ prediction		0–25%	26–50%	51–75%	76–100%
Risk of relapse without chemotherapy	30.4 (16.2)	43.4	**45.3**	10.1	1.1
Risk of relapse with chemotherapy	13.3 (8.4)	**91.4**	8.6	–	–
Risk of severe toxicity	9.8 (7.4)	**96.6**	3.4	–	–
Breast cancer patients’ prediction					
Risk of relapse without chemotherapy	78.6 (20.4)	1.7	19.1	**40.2**	39.0
Risk of relapse with chemotherapy	29.6 (20.4)	**72.5**	22.9	2.1	2.5
Risk of severe toxicity	61.0 (22.8)	14.2	**40.0**	32.5	13.3

*Bold, *p* < 0.05.*

Breast cancer patients’ prediction of risk of recurrence without chemotherapy was 78.6% (40.2% felt it was high), while they estimated a 29.6% risk of relapse with chemotherapy (72.5% thought it was very low). The estimated risk of suffering severe toxicity was 61% (40% of patients deemed that it was low).

Oncologists and patients differed significantly in their prediction of relapse without chemotherapy (χ^2^ = 15.901, *p* = 0.006) and with chemotherapy (χ^2^ = 9.702, *p* = 0.021) and in their estimation of severe toxicity (χ^2^ = −12.120, *p* = 0.007). For all three situations, patients believed they were at higher risk than did oncologists, overestimating the risk of recurrence with and without chemotherapy, as well as their risk of severe side effects if they received chemotherapy.

### Estimation of Risk of Recurrence and Toxicity by Stage

For patients with stages IB, II, and III disease, oncologists’ prognoses regarding risk of relapse without chemotherapy were 25.8, 31.7, and 42%, respectively. They estimated risk of recurrence with chemotherapy to be 10.3, 13.6, and 23.9%, respectively. The risk of severe toxicity with chemotherapy was believed to be 9.1, 10.2, and 9.9%, respectively see (Table 2).

**TABLE 2:** Prognostic predictions about risk of relapse and toxicity by stage.

Variables	Stage IB	Stage II	Stage III	*F*	*p*	Effect size η^2^
				
	M (SD)	M (SD)	M (SD)			
Oncologists’ prediction						
Risk of relapse without chemotherapy	25.8 (13.1)	31.7 (17.1)	42.0 (15.2)	11.529	**0.001**	**0.080**
Risk of relapse with chemotherapy	10.3 (5.7)	13.6 (8.4)	23.9 (9.5)	30.852	**0.001**	**0.189**
Risk of severe toxicity	9.1 (5.5)	10.2 (9.0)	9.9 (3.5)	0.778	0.460	–
Breast cancer patients’ prediction						
Risk of relapse without chemotherapy	72.4 (21.0)	81.8 (19.5)	86.9 (16.6)	7.958	**0.001**	**0.063**
Risk of relapse with chemotherapy	29.1 (20.9)	28.4 (19.7)	38.0 (21.1)	2.224	0.110	–
Risk of severe toxicity	58.5 (22.0)	61.9 (22.5)	65.2 (22.8)	1.000	0.369	–

*Bold, *p* < 0.05.*

Patients with breast cancer stages IB, II, and III predicted their risk of recurrence without chemotherapy to be 72.4, 81.8, and 86.9%, respectively. They estimated their risk of relapse with chemotherapy to be 29.1, 28.4, and 38%, respectively, and of suffering severe toxicity to be 58.5, 61.9, and 65.2%, respectively.

Clinicians predicted risk of relapse differently depending on tumor stage, estimating greater risk without chemotherapy (*F* = 11.529, *p* = 0.001, η^2^ = 0.080) and with chemotherapy (*F* = 30.852, *p* = 0.001, η^2^ = 0.189) for individuals with higher stages. The same trend was apparent in patients’ prediction of risk of relapse without chemotherapy by stages (*F* = 7.958, *p* = 0.001, η^2^ = 0.063). No significant stage-based differences were observed in patients’ predictions of risk of relapse with chemotherapy or of severe toxicity.

### Factors Modulating Estimated Risk

There is a significant correlation between oncologists’ age and years of experience and their risk prediction. Young doctors and those with fewer years of experience prognosticate greater risk of relapse than older, more veteran physicians both without chemotherapy (*r* = −0.267, *p* < 0.001; *r* = −0,256, *p* < 0.001, respectively) and with chemotherapy (*r* = −0.273, *p* < 0.001; *r* = −0.256, *p* < 0.001, respectively). The same was seen with respect to risk of severe toxicity (*r* = −0.213, *p* < 0.001; *r* = −0.188, *p* < 0.001, respectively). However, participants’ risk prediction did not correlate with doctors’ age, years of experience, or patients’ age (see Table 3).

**TABLE 3:** Correlations between prognostic prediction and sociodemographic variables.

	Oncologists’ prediction			Breast cancer patients’ prediction		
Variables	Relapse without chemo	Relapse with chemo	Toxicity	Relapse without chemo	Relapse with chemo	Toxicity
Oncologists’ age	−0.267**	−0.273**	−0.213**	0.076	−0.088	−0.013
Years of experience	−0.256**	−0.256**	−0.188**	0.064	−0.083	−0.012
Patient’s age	−0.011	−0.054	−0.070	0.101	0.099	−0.022
SDM Oncologist	−0.079	−0.160*	−0.029	–	–	–
SDM patient	–	–	–	0.035	−0.054	−0.228*
BSI psychological distress	–	–	–	0.026	0.206**	0.096

*p < 0.01; **p < 0.001.

SDM-Q-Doc and Q-9 correlated significantly with some predictions. The higher the estimated risk of relapse with chemotherapy, the less satisfied the oncologist was with SDM (*r* = −0.160, *p* < 0.01). Similarly, patients were less satisfied with SDM when their risk of severe toxicity was higher (*r* = −0.228, *p* < 0.01).

There is a positive, significant correlation between psychological distress (BSI-18) and patient prognosis of greater risk of recurrence with chemotherapy (*r* = 0.206, *p* < 0.001).

## Discussion

Overall, patients’ predictions of risk their cancer recurring with and without chemotherapy tended to be higher than oncologists’. Moreover, while physicians estimated greater risk of relapse as disease stage increased, patients only exhibited this tendency when they predicted risk of recurrence without chemotherapy, trusting in their treatment regardless of their initial medical situation. In general, doctors considered that the risk of relapse with and without chemotherapy was low and very low, respectively, whereas patients felt that their risk with chemotherapy was very low and high without chemotherapy, respectively. The same trend was observed for risk of toxicity. The literature points toward oncology patients being more optimistic than oncologists in the context of advanced cancer (Robinson et al., 2008); nonetheless, we have only found one study that examines doctors’ and cancer patients’ perceptions in the early stages of the disease. An old study reports that while patients and oncologists coincided insofar as risk of recurrence without adjuvant treatment is concerned, such was not the case with adjuvant treatment (60% patients overestimated their possibilities of being cured with adjuvant treatment by 20% or more vs. their oncologists) or with respect to treatment-associated risks. These results, which differ from ours, are not directly comparable, given that therapies, survival, and physician–patient communication have changed tremendously over time. Focusing on the patients, we detected a sizable difference between the perception of risk of relapse with chemotherapy (high) and without (very low). Patients’ perception of less risk of relapse with or without adjuvant treatment is disproportionate and unrealistic in terms of the expected benefit and is also seen in a classic study, where expectations of a 79% decrease in the risk of recurrence with adjuvant chemotherapy were observed (Ravdin et al., 1998). We believe that three different factors may influence our results: (1) doctor–patient communication, which we consider should be less paternalistic and more patient-focused; (2) patients’ knowledge about cancer; and (3) interpersonal relations with other agents, notably the media and patient associations.

Several studies refer to the importance of physician–patient communication (Gramling et al., 2016; Bos-van den Hoek et al., 2019; Trant et al., 2019). Likewise, some classic studies have determined that the discrepancy between doctors’ and patients’ expectations may be due to physicians failing to provide quantitative data (Siminoff et al., 1989; Loprinzi et al., 1994). Although doctors are increasingly furnishing more quantitative data regarding the disease (Belkora et al., 2009; Zikmund-Fisher et al., 2016), most patients continue to estimate their risk of recurrence and treatment-associated risk inaccurately (Belkora et al., 2009). Consequently, variables such as level of education, psychological status, or patients’ familiarity with medical concepts may affect their estimations. Similarly, some authors suggest that it is difficult for patients to understand the technical and scientific aspects of chemotherapy (Wakiuchi et al., 2019), which ultimately leads to knowledge based on their own experience in which aspects such as their vision of the disease, their relatives’ and acquaintances’ experiences in the same circumstance, their own coping style, or the confidence they have in themselves to confront their cancer all take precedence (Slevin et al., 1990; Wakiuchi et al., 2019; Bos-van den Hoek et al., 2019). The lack of information concerning the medical condition and/or treatment is one of the leading causes of patients’ dissatisfaction (Calderon et al., 2018). In fact, at present, most want to receive more information, as well as to participate more in the decision-making process, albeit the proportion of people who prefer a more active role differs across countries (Calderon et al., 2018). A survey conducted in eight European countries revealed that patients wanted to participate in decision making, although their expectations about their involvement in healthcare decisions differed significantly across countries; for example, in Spain, participants preferred a more paternalistic model than in Switzerland or Germany (Elwyn et al., 2014).

As for cognitive aspects, the fact that they had only learned of their cancer diagnosis a few weeks earlier may have led society’s fear of cancer to prevail, as it is generally associated with death (Wakiuchi et al., 2019), as well as the feeling that their very survival was at stake (Mackillop et al., 1988). In our study, we believe that the scenario of tremendous uncertainty and possible physical discomfort due to recent surgery and current recovery, together with initiating a new treatment, may have magnified patients’ estimation of risk of relapse and toxicity.

Other studies also point to the effect of other sources of information, such as relatives, cancer survivors, support groups, non-physician healthcare personnel, and educational material that could modify patients’ perception of risk of relapse (Adamson et al., 2019).

Turning our attention to the physician cohort, prediction of risk of relapse, with and without chemotherapy, and of severe toxicity was related to oncologists’ age and years of experience. This is consistent with other studies, where these doctor-related variables contribute to shaping their prognosis (Taniyama et al., 2014). On the other hand, they feel that SDM is more difficult when they perceive a higher risk of relapse. Smith et al. (2013) report that discussing uncertainty and risks challenges patient–clinician communication; therefore, when the physician finds himself/herself in a complex situation, he/she may feel more uncomfortable with SDM.

As for patients, those suffering greater psychological distress predicted higher risk of relapse with chemotherapy. This reveals a negative, defeatist attitude, without losing sight of the numerous studies that indicate that depression and anxiety correlate with a higher risk of mortality in cancer patients (Shim et al., 2020). This illustrates the need to detect and support these individuals to relieve their psychological distress, which will not only affect their quality of life, but may also improve their prognosis. In contrast, patients who are more satisfied with SDM estimated a lower risk of severe toxicity, which may indicate that a good interview bolsters their confidence in treatment and gives them a greater perception of safety. Hjerl et al. (2003) found that treatment side effects influence patients’ acceptance of treatment. As previously laid out, most patients were willing to accept intensive chemotherapy for even a slight chance of benefit (Slevin et al., 1990).

This study’s findings should be considered in conjunction with its limitations. First, the present study was cross-sectional in nature; therefore, it was not possible to determine the directionality of the relationships observed. Future studies should explore perceptions of risk of relapse and severe toxicity after adjuvant chemotherapy is completed, with an end to assessing whether these perceptions are changing over time and how variables relate to one another. Second, patients’ and oncologists’ responses have been collected by means of questionnaires, which, on the one hand, may have limited the spectrum of answers and, on the other, hindered delving into personal motivations and comprehension. Third, the results obtained in this study are specific for patients who underwent surgery with curative intent for stage I, II, or III breast cancer, making it difficult to extrapolate to metastatic disease and other types of cancer given the specificities of this patient cohort. Fourth, in all cases, the indication for adjuvant chemotherapy was based on international clinical guidelines, and all agreed to receive it; consequently, we do not know to what degree this decision may have influenced patients’ predictions of their risk of relapse without chemotherapy, as a variable they may not have deemed realistic. Finally, it would be advisable to enlarge the sample of oncologists in the future.

As for the clinical implications of these findings, SDM about adjuvant treatment and patients’ understanding of cancer prognosis are complex issues; hence, it may be necessary to ascertain each individual’s perception and communicate information over consecutive visits. Furthermore, improving physician–patient communication in pursuit of more realistic expectations regarding risk of relapse and toxicity could lessen anxiety, depression, and fear of treatment, thereby enhancing patients’ quality of life.

## Data Availability Statement

The datasets generated for this study are available on request to the corresponding author.

## Ethics Statement

This study was approved by the Ethics Committees of all study sites and the Spanish Agency of Medicines and Medical Devices (AEMPS). The patients/participants provided their written informed consent to participate in this study.

## Author Contributions

CC and PJ-F: study conception design. LC-S, CC, and PJ-F: data analysis, interpretation, and drafting of the manuscript. All authors contributed to data collection and critical revision of the manuscript.

## Conflict of Interest

The authors declare that the research was conducted in the absence of any commercial or financial relationships that could be construed as a potential conflict of interest.

## Footnotes

1 www.neocoping.es
